# Open plains are not a level playing field for hominid consonant-like versus vowel-like calls

**DOI:** 10.1038/s41598-023-48165-7

**Published:** 2023-12-21

**Authors:** Charlotte Gannon, Russell A. Hill, Adriano R. Lameira

**Affiliations:** 1 Department of Psychology, University of Warwick, Coventry, UK; 2 Department of Anthropology, Durham University, Durham, UK; 3 Primate and Predator Project, Soutpansberg Mountains, Thohoyandou, South Africa; 4 Department of Biological Sciences, University of Venda, Thohoyandou, South Africa

**Keywords:** Palaeoecology, Evolution, Cultural evolution, Evolution of language

## Abstract

Africa’s paleo-climate change represents an “ecological black-box” along the evolutionary timeline of spoken language; a vocal hominid went in and, millions of years later, out came a verbal human. It is unknown whether or how a shift from forested, dense habitats towards drier, open ones affected hominid vocal communication, potentially setting stage for speech evolution. To recreate how arboreal proto-vowels and proto-consonants would have interacted with a new ecology at ground level, we assessed how a series of orangutan voiceless consonant-like and voiced vowel-like calls travelled across the savannah. Vowel-like calls performed poorly in comparison to their counterparts. Only consonant-like calls afforded effective perceptibility beyond 100 m distance without requiring repetition, as is characteristic of loud calling behaviour in nonhuman primates, typically composed by vowel-like calls. Results show that proto-consonants in human ancestors may have enhanced reliability of distance vocal communication across a canopy-to-ground ecotone. The ecological settings and soundscapes experienced by human ancestors may have had a more profound impact on the emergence and shape of spoken language than previously recognized.

## Introduction

Over the last 17 million years, continental tectonic movement^1^ combined with global cooling and aridification resulted in the spread of grasslands across Eurasia and Africa^2^, ultimately setting the stage for the emergence of the human lineage^3,4^. Throughout these continents’ landmass, dense mixed forests^5^ gave way to generally open and drier habitats as new regional but complex patterns of wet-dry cycles became established^6–8^. These extreme climate changes imposed new ecological conditions for hominid survival, which resulted in major great ape extinctions starting at 9.5 million years ago in Eurasia and Africa^9–12^. While the transformations imposed on hominid anatomy and behaviour can be directly reconstructed from archaeological sites^4,13^ or inferred from paleo-ecology^9,14,15^, one major component of human evolution is unrecoverable from the fossil record—the evolution of spoken language and its precursor, vocal signals.

Paleontological evidence can help understand the anatomical changes that facilitated the expression of language through speech, such as larynx’s descent^16^, air-sacs’ recession^17–19^, thoracic innervations’ increase^20,21^ and mandibular transformations^22,23^. However, these structures are remote surrogates of the signals used to de facto communicate vocally, and ultimately, to articulate (proto)language. These structures have also been tempered by major selective forces driving other changes in human anatomy, such as head and vocal tract re-organization due to a rising bipedal posture^24^ and diet changes^23^, overall, making it difficult to parse language-causes versus language-consequences based on anatomy or the fossil record alone.

Being our closest living relatives, nonhuman great apes represent extant models of hominids’ ancestral biology and behaviour^10^, including homologous sound production^25^ and sound perception capacities^26^. They provide an alternative and potent means to explore why, among its contemporary and sympatric species, only the repertoire of ancient hominids took a turn towards spoken language in the wake of continent-wide ecological transformations, from densely forested habitats to drier and more open soundscapes.

### Great ape proxies of proto-vowels and proto-consonants

All great ape genera produce voiceless consonant-like calls^25,27–35^ and voiced vowel-like calls^36–39^, in direct articulatory and acoustic homology with all the world’s languages. *Pan* (chimpanzees and bonobos) and *Pongo* (orangutans), can produce, respectively, one and both of these call categories at speech-like rhythm^32,40^. Given that each and every spoken language is composed by consonants and vowels that combine to compose a language’s words and sentences, great ape consonant-like and vowel-like calls offer desirable proxies of the two putative speech precursor elements.

Among great apes, orangutans exhibit a particularly large consonant-like repertoire^41^ that they combine with various vowel-like voiced calls to produce syllable-like combinations^36^ in complex sequences^28,42,43^. Consonant-like and vowel-like calls exhibit unique acoustic profiles that result from distinct mechanics and control, and in orangutans, both have been shown to be under fine motor laryngeal^44–46^ and supra-laryngeal control^30,31^. Similarly to the speech sounds, both call categories can occur as population-specific traditions in orangutans instead of representing calls that are universal in the species, and their articulation and acoustics are also socially moulded^47–52^. In addition, wild orangutans use these combinations as a canvas for the expression of high cognitive processes, such as tool-modulated vocal deception^28^ and vocal communication about past events^53^.

To address the role of paleo-climate change on spoken language evolution, African apes, for instance, could allow comparison of the same vowel-like call between forest and savannah populations of the same species or genus. However, orangutans are the only great ape currently known to produce syllable-like call combinations in the wild, making these structures the only extant models that can be considered as combinatorial homologues of proto-linguistic structures in human ancestors. Furthermore, orangutans are the most arboreal of all great apes^54^, providing a rare opportunity to explore the “canopy-to-ground” ecological shift experienced by ancient hominids and human ancestors. Indeed, cumulating evidence shows that human ancestors were more arboreal than traditionally appreciated, and more than extant African apes^41,55–58^.

### Re-staging the ecology of spoken language evolution

By taking advantage of orangutans’ arboreal consonant-like and vowel-like calls and moving them to an open landscape setting, we can recreate, as close and realistically as it is possible today, the scenario in the Middle and Late Miocene (16–5.3 mya) when hominids transitioned down from trees to an open landscape. This is not to suggest that modern day consonants, vowels, or syllables directly descended from orangutans^58,59^. Instead, orangutans’ speech-like calls and combinations offer extant homologues to study extinct proto-linguistic units and structures.

For spoken language to evolve, consonant-like and vowel-like calls had to marry to give birth to proto-syllables, -words and -sentences. While vowel-like calls are prototypical to all nonhuman primates and well represented in the call repertoires of all great apes, consonant-like calls are predominantly found in arboreal versus ground-dwelling great apes^41^. This implies that, whenever and wherever human ancestors descended from trees during the Mid and Late Miocene, they brought with them an arboreal repertoire, predicted to have been rich in consonant-like calls, in addition to primates’ prototypical vowel-like repertoire. Understanding what this transition meant for the use of the two foundational elements for speech is, hence, critical for an authentic reconstruction of the speech’s evolutionary process and origin of language-able apes.

**Figure 1:**
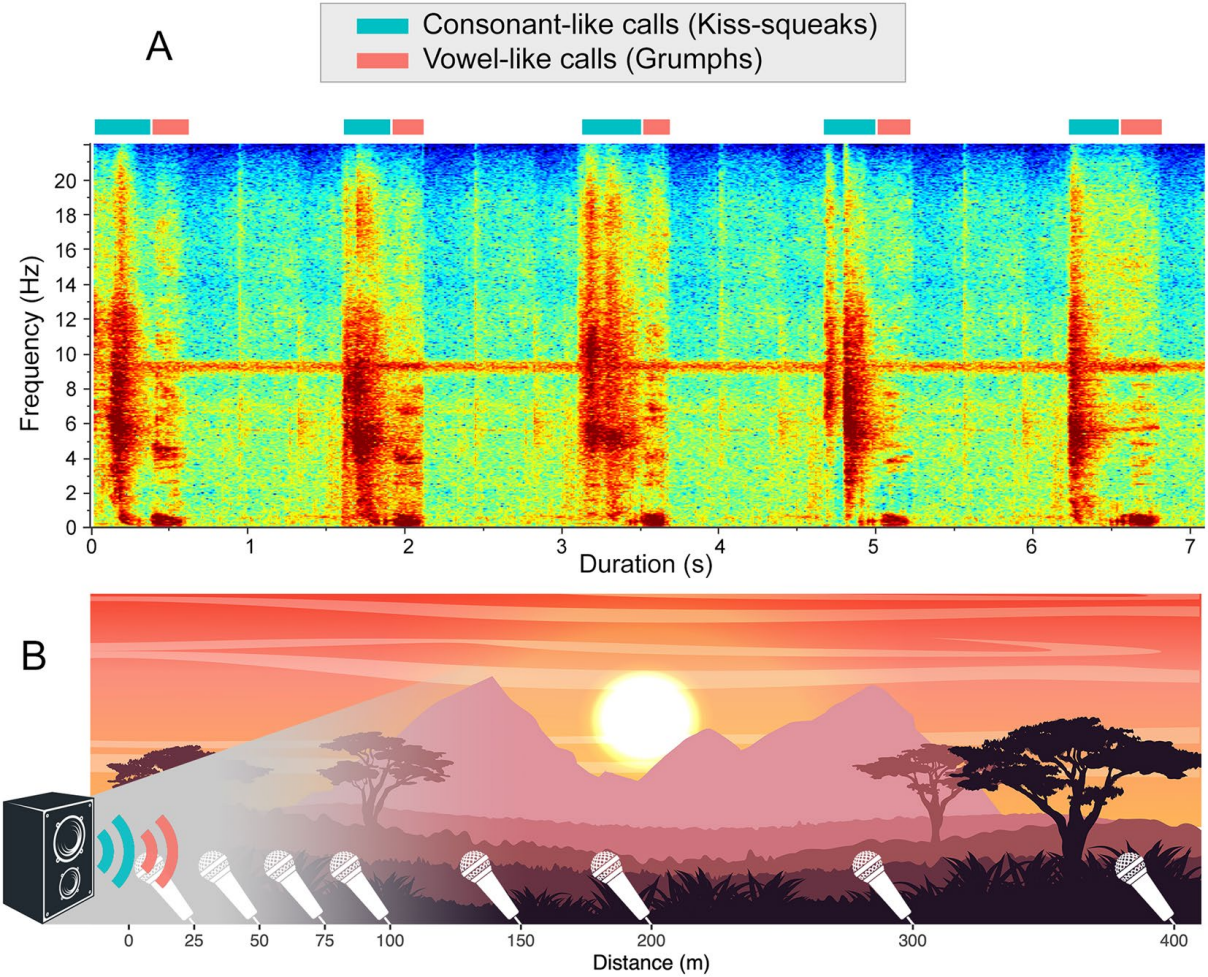
Spectrographic representation of orangutan consonant-like and vowel-like calls (above) and experimental set up (below).

To shed new light on the ecology of spoken language evolution and the most dramatic climate transition known to have occurred alongside the divergence of human ancestors from other hominid lineages, we conducted playbacks of orangutan voiceless consonant-like ‘kiss-squeaks’^36^ produced in combination with voiced vowel-like ‘grumphs’^36^ across an African savannah. We then quantified their respective acoustic performance by recording played back calls at increasing distances up to 400 m (Fig. 1, Supplementary data 1). Played back calls were originally recorded across various individuals, various populations and under three different contexts (towards observers, towards a white predator-model and towards a tiger-patterned predator-model)^43,49,60,61^. Previous research has established that orangutan consonant-like and vowel-like calls can both be information-dense^62^ and that they both broadcast and transmit information successfully across a dense rainforest up to 100 m distance^63^. However, how the two call categories behave and interact in open landscapes with fewer to no physical obstructions against signal transmission, such as the savannah, remains unknown. The classic inverse square law of sound propagation for pure tones predicts that low frequencies degrade less over distance than higher frequencies. Accordingly, vowel-like calls, which typically show lower frequency contours than consonant-like calls, would be theoretically predicted to carry further and better. Nonetheless, previous bioacoustics research has shown that theoretical models are poor substitutes for real, living biological calls with acoustic profiles more complex than pure tones^63^. The question of whether consonant-like and vowel-like calls behaved equally in the emergent ecology of open landscapes during human evolution remains, thus, open.

## Results

**Figure 2:**
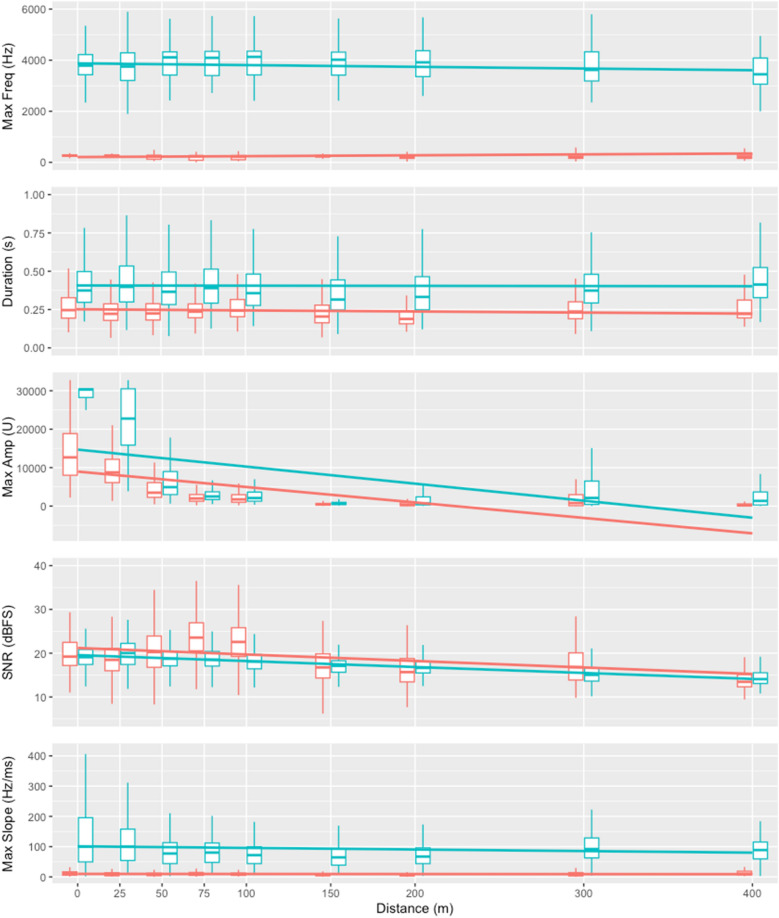
Acoustic performance over distance. Box plots represent median and 25–75% interquartile range, whiskers represent lower/highest value within 1.5 times interquartile range below/above, outliers omitted strictly for visual clarity (they were retained in all analytical procedures). Linear trend lines represented across distance are for visual aid only (based on raw data, not model projections).

The acoustic performance of both voiceless consonant-like kiss-squeaks (N = 487 at 0 m distance) and voiced vowel-like grumphs (N = 487 at 0 m distance) changed significantly over the 400 m of savannah (Fig. 2, Supplementary data 2). That is, delta time, max frequency, max slope, signal-to-noise ratio (SNR) and max amplitude all significantly degraded (or presented absence thereof) with increasing distance for both kiss squeaks (LMM ANOVA, p < 0.001 for all parameters; Supplementary data 2) and grumphs (LMM ANOVA, p < 0.001 for all parameters; Supplementary data 3).

In addition to an effect over distance, population and/or context had a significant effect on several acoustic parameters for kiss squeaks (LMM ANOVA, delta time/population: p < 0.02; delta time/context: p < 0.001; max frequency and max slope/context: p < 0.001; SNR/population: p = 0.003; SNR/context: p = 0.0037; max amplitude/context: p < 0.001; Supplementary data 2). Context also had a significant effect on several acoustic parameters for grumphs (LMM ANOVA, delta time and max slope: p < 0.001; max amplitude: p = 0.003; Supplementary data 3).

**Figure 3:**
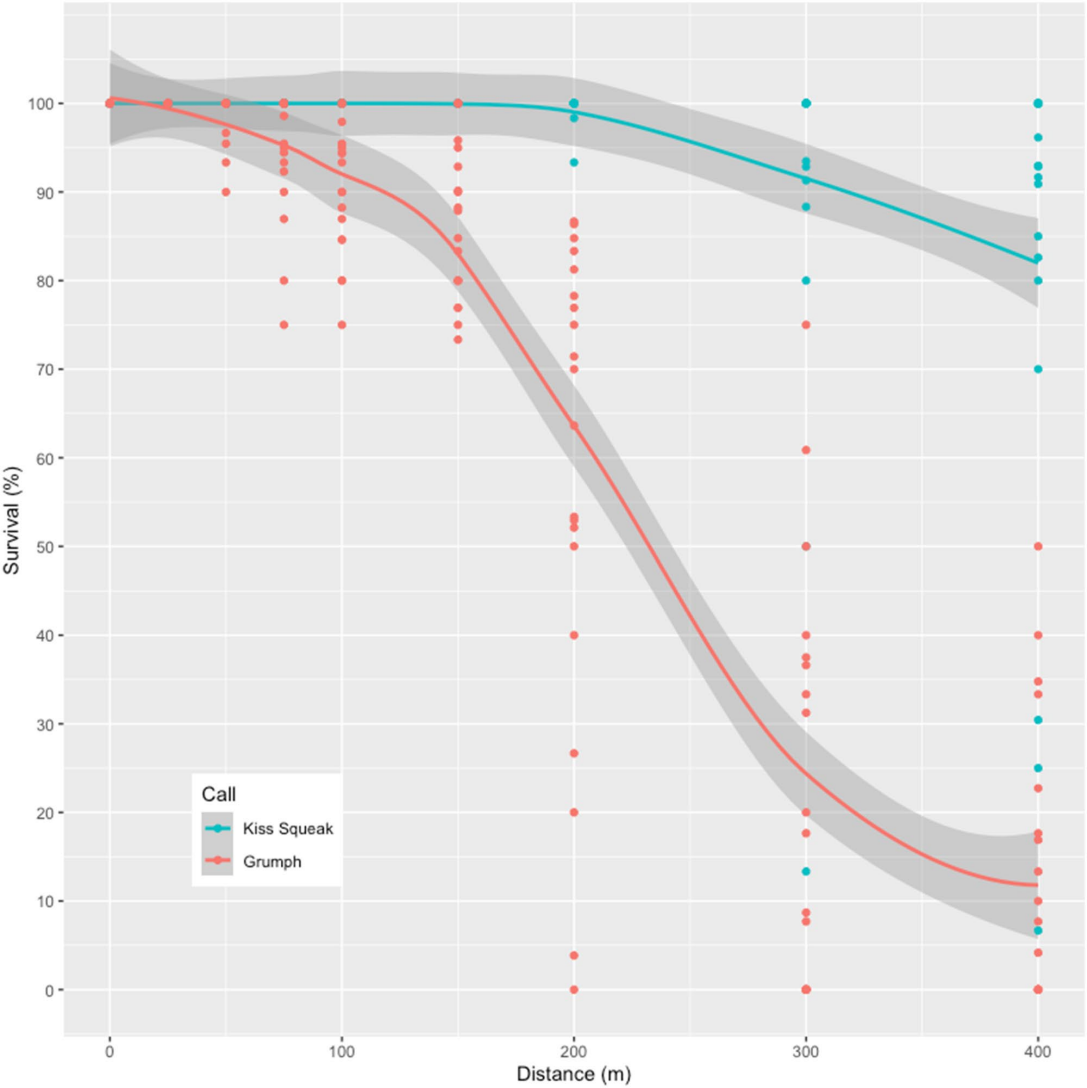
Acoustic survival over distance. Perceptibility of consonant-like and vowel-like calls across increasing savannah distances up to 400 m, 95% confidence interval (shaded area).

In terms of overall detection or “acoustic survival”—that is, whether calls were audibly perceptible and visible on the spectrogram or not—vowel-like calls’ survival was significantly compromised and significantly lower than consonant-like calls from 100 m onwards. That is, there was no overlap between bands of 95% confidence interval of both calls after this distance (Fig. 3). After a sharp decay around 125 m away, less than 20% of vowel-like call survived up to 400 m distance. Conversely, consonant-like calls exhibited only a modest decrease in survival from 250 m onwards, with ~ 80% of the calls surviving (i.e., remaining perceptible) up to 400 m distance.

## Discussion

We established that consonant-like and vowel-like calls produced in combination by a wild arboreal great ape degrade differently across a savannah open plain. Notably, orangutan vowel-like grumphs exhibited a significantly lower acoustic performance than orangutan consonant-like kiss-squeaks, with consonant-like calls significantly outperforming their vowel-like counterparts beyond distances of 100 m. At 400 m vowel-like calls were largely inaudible (< 20% survival rate), whereas most consonant-like calls were still largely detectable and perceptible (> 80% survival rate). Additional population and context effects were not unexpected based on known geographic and contextual variation encoded in orangutan vocal signals^62^ and primate call communication more generally.

Our setup involved a *series* of calls played back every few seconds for roughly 15 min. While this was ideal to assess sound propagation over distance, as classically used in bioacoustics^61,63–66^, the repetition of calls probably inflated the chances of call detection during the phase of extraction of acoustic parameters. This may have particularly inflated the detection of faint or partly inaudible vowel-like calls, given they had a rapid acoustic degradation and poor survival over distance (i.e., acoustic survival = 0% at 200, 300 and 400 m; Fig. 3; Supplementary data 3). Conversely, repetition of consonant-like calls seems to have been largely redundant given that their acoustic survival rates were largely immune to distance. If we had played back solo or short call series (as kiss-squeaks and grumphs frequently occur naturally), it could have been possible that vowel-like calls would be fully imperceptible beyond 125 m.

### Implications for signal and information theory

Results show that the law of sound propagation for pure tones, which predicts that lower-frequency sounds (characteristic of voiced vowel-like calls) travel farther than higher-frequency (characteristic of voiceless consonant-like calls)^67^, does not apply to natural calls. Natural calls are not pure tones, but instead, composed by a varied mix of wide sound bands, particularly voiceless calls. These are typically noisy and present, hence, a wide spread of acoustic energy across the frequency spectrum. Natural calls with complex and composite acoustic profiles behave differently as a whole than defined, “clean” frequencies. This made our results the opposite of what would be theoretically expected for pure tones; the acoustic survival of higher-frequency consonant-like calls was higher than lower-frequency vowel-like calls. This speaks to the importance of using great ape calls as extant models for spoken language origin and evolution, despite the common use of purely theoretical, computational, and/or mathematical models in the field^68^.

### Evolutionary implications

Results show that paleo-climate change during the Middle and Late Miocene put forth new selection forces for hominid consonant-like versus vowel-like calls. Where dense forests had offered equivalent performance for both call categories^63^, the newly emerging dry and open landscapes offered superior transmission efficiency to consonant-like calls over mid and long distances. Until the time of these ecological transformations, primates in general, and hominids in particular, had relied for millions of years on the production of repetitive, strenuous, and vigorous loud vocal displays for distance communication, composed by voiced vowel-like calls^69^. In the new ecological settings, hominids now had an element available—consonant-like calls—in their vocal repertoire with enhanced perceptibility, if and when required.

Enhanced perceptibility of consonants versus vowels at the earliest stages of speech evolution could help to start explaining some features of modern spoken languages. For instance, consonants can act as natural cues in speech, preferably used by language learners for perceptually breaking sentences and inserting processing pauses where there are none^70^. Also, during the babbling stage of an infant’s language acquisition and development, the larger the range of consonants they are exposed to, the earlier they will typically begin to babble and reach speech development milestones^71^. Infants also learn to rely on consonants more than vowels to identify words after their first year^72^. Consonants and vowels then continue to play different roles for language users as adults; consonants are relied on to extract cues primarily related to semantic information, whereas vowels are relied on to extract cues primarily related to syntax^73^. All these linguistic phenomena depend on heightened conspicuity of consonant sounds. Accordingly, it is possible that consonants’ growing role in hominid vocal communication started to play out in the new land- and soundscapes that human ancestors encountered in the wake of paleo-climate change. The ecology of ancient hominids may have moulded human modern verbal behaviour to a larger extent than hitherto appreciated.

## Methods

### In brief

Consonant-like and vowel-like calls that had been previously recorded in the form of syllable-like combinations from wild orangutans were played back across a savannah habitat in South Africa and re-recorded over increasing distance. Five acoustic parameters were extracted from the recordings at distance intervals from zero to 400 m away. The degradation of these parameters was used to evaluate the acoustic performance of consonant-like and vowel-like calls. Individual, contextual, and geographical variation among consonant-like and vowel-like calls, and the capacity of a receiver to assign a call to its correct class, was used to evaluate information broadcast over distance.

### Study site

Playback experiments were conducted at Lajuma in the western Soutpansberg Mountain Range, Limpopo Province, South Africa (23°06*′*45.14*′′* S, 29°11′37.10*′′ *E). The playback took place in a grassland with woodland patches at elevation of 1420m^74^. The wider Soutpansberg’s geography presents a complex environment, from mist-belt forest groups and closed woodlands, to bushveld complex and rocky mountainous regions^75^.

### Data collection

The playback call set was made up of 487 calls from populations of Sumatran (*Pongo abelii*) and Bornean orangutans (*Pongo pygmaeus*); playbacks included calls from 20 different individuals, three different contexts, and three different geographical populations^63^. The recordings were collected from three research stations: Gunung Palung and Tuanan in West and Central Kalimantan, Indonesian Borneo, and in Sampan Getek in North Sumatra, Indonesia. All kiss-squeaks and grumphs selected for playback were produced in kiss-squeak + grumph call combinations. In preparation for playback, all call combinations were set to the same peak amplitude using Raven interactive sound analysis software (v. 1.6, Cornell Lab of Ornithology, Ithaca, New York). In other words, we ensured that the loudest point of each call combination was as loud as the others. This preserved the natural difference in loudness between the call types within the same combination.

The playback speaker was set at 0 m and calls were re-recorded every 25 m up to 400 m along a transect across flat and dry terrain. All playbacks were conducted on the same morning and meteorological conditions remained consistent during the experiment. There had been no heavy wind or rainfall in the previous 48 h of recording. Theoretically, wind and rain were assumed to be adverse to sound transmission across habitats, including forests for example^63^, not just open habitats. Playbacks were conducted once per distance using a Marantz Digital Recorder PMD-660 (D&M Holdings, Kawasaki, Japan) connected to a Nagra DSM speaker (Audio Technology Switzerland S.A., Romanel, Switzerland). The base of the speaker was set between 1 and 1.5 m from the ground. The playbacks were re-recorded using a ZOOM H4next Handy Recorder (ZOOM Corporation, Tokyo, Japan) connected to a RØDE NTG-2 directional microphone (RØDE LLC, Sydney, Australia). Audio data was recorded using the WAVE PCM format at 16 bits. The mic was set parallel to the ground from 1 to 1.5 m. All volume settings for the mic and speakers remained equal for all distances.

### Data measurements

We extracted acoustic parameters per call across each distance of 25, 50, 75, 100, 150, 200, 300 and 400 m away from the speaker. Data from 0 m was extracted from the original playback call set. Acoustic parameters per call were extracted by manually drawing a selection box in Raven around a calls or whichever call components were still visible. If no trace of a call was visible in the spectrogram, no selection was drawn and no parameters were extracted, given that a call was effectively inaudible in these cases. A call was then considered to not have survived at that distance. All selections were drawn by the same experimenter to avoid inter-observer biases. Five acoustic parameters were measured from all calls using Raven interactive sound analysis: delta time (s), max frequency (Hz), peak frequency counter slope (Hz/ms), signal-to-noise ratio (SNR) (dB FS) and max amplitude (u) (Supplementary data pages 1–16). Delta time is the time difference between the offset and onset of each call. Max frequency is the frequency with the highest amplitude in a call. PFC max slope is the increase or decrease of the slope for each call. SNR is the signal power from the call compared to background. Max amplitude is the maximum power of the sound wave for each call. These parameters were used as previous research has demonstrated that they are able to provide strong descriptors of acoustic and informational context^49^. Each of the 5 parameters could be extracted from both the kiss squeaks and grumphs, enabling direct comparison between the acoustic and informational performance across distances for both call types.

### Data analyses

To assess acoustic performance, Linear Mixed Models (model type: III sum of squares; test model terms: Satterthwaite) were conducted using JASP (v. 0.15). One model was generated for each acoustic parameter (× 5; i.e., time, max freq, max slope, SNR and max amp) under both call type conditions (× 2; i.e., kiss-squeak and grumph), resulting in 10 models in total. For each model, the acoustic parameter was inserted as dependent variable (N = 4278 kiss-squeaks, N = 3302 grumphs). Distance (ordinal: 0, 25, 50, 75, 100, 150, 200, 300, 400 m), context (three levels: calls produced towards observers, towards a white predator-model, towards a tiger-patterned predator-model) and geographical population (three levels: Tuanan, Gunung Palung, Sampan Getek) were inserted as fixed effect variables. Context and population were included in analyses of acoustic performance to maintain the same call sample used for information performance analyses.

Additionally, name of individual (N = 20) was inserted as random effect, as some individuals were re-used across the 3 different contexts. Call ID (33 levels) was inserted as random effect, given that the same calls were re-recorded per distance. LMM graphs were created in R using ggplot2 and gridExtra. For grumph’s max frequency, we obtained a warning that model fit was singular, and so, for this model, we removed call ID as random effect, which yielded the same qualitative results. To graphically depict the “acoustic survival” of each call type, we used ggplot2 in R to render the percentage of calls heard per distance.

### Supplementary Information

Supplementary Information 1.

Supplementary Information 2.

Supplementary Information 3.

## Data Availability

All data needed to evaluate the conclusions in the paper are present in the paper and/or the electronic supplementary materials. Audio files are available on the Open Science Framework (https://osf.io/6hkaq/?view_only=048983a0c3744cb9814431e1b084dc2c). Additional data may be requested from the corresponding author, CG, upon reasonable request.
