# Supplementary material for: Open plains are not a level playing field for hominid consonant-like versus vowel-like calls
Source: Sci Rep. 2023 Dec 21;13:21138. doi: 10.1038/s41598-023-48165-7 (PMC10739746; doi:10.1038/s41598-023-48165-7)
Supplement: Supplementary file 1 — Supplementary Information 1. [file 41598_2023_48165_MOESM1_ESM.pdf]

Table 1: Descriptive Statistics for Call Types

Descriptive Statistics

	Name of Ind		Population		Paradigm		Context	
	Grumph	Kiss Squeak	Grumph	Kiss Squeak	Grumph	Kiss Squeak	Grumph	Kiss Squeak
Valid	3315	4280	3315	4280	3315	4280	3315	4280
Missing	0	0	0	0	0	0	0	0

Frequency Tables

Frequencies for Name of Ind

Call type	Name of Ind	Frequency	Percent	Valid Percent	Cumulative Percent
Grumph	Aminah	24	0.724	0.724	0.724
	Asny	29	0.875	0.875	1.599
	Bintang	113	3.409	3.409	5.008
	Desy	489	14.751	14.751	19.759
	Indi	100	3.017	3.017	22.775
	Inul	341	10.287	10.287	33.062
	Jinak	300	9.050	9.050	42.112
	Juni	472	14.238	14.238	56.350
	Kasi	107	3.228	3.228	59.578
	Keri	424	12.790	12.790	72.368
	Kondor	178	5.370	5.370	77.738
	Male	72	2.172	2.172	79.910
	Mindi	118	3.560	3.560	83.469
	Pensi	85	2.564	2.564	86.033
	Sumi	145	4.374	4.374	90.407
	Walimah	89	2.685	2.685	93.092
	uf2	65	1.961	1.961	95.053
	uf3	61	1.840	1.840	96.893
	uf5	73	2.202	2.202	99.095
	uf6	30	0.905	0.905	100.000
	Missing	0	0.000		
	Total	3315	100.000		
Kiss Squeak	Aminah	31	0.724	0.724	0.724
	Asny	45	1.051	1.051	1.776
	Bintang	153	3.575	3.575	5.350
	Desy	634	14.813	14.813	20.164
	Indi	135	3.154	3.154	23.318
	Inul	428	10.000	10.000	33.318
	Jinak	403	9.416	9.416	42.734
	Juni	587	13.715	13.715	56.449
	Kasi	124	2.897	2.897	59.346
	Keri	523	12.220	12.220	71.565
	Kondor	189	4.416	4.416	75.981
	Male	90	2.103	2.103	78.084
	Mindi	162	3.785	3.785	81.869
	Pensi	117	2.734	2.734	84.603
	Sumi	233	5.444	5.444	90.047
	Walimah	107	2.500	2.500	92.547
	uf2	90	2.103	2.103	94.650
	uf3	85	1.986	1.986	96.636
	uf5	100	2.336	2.336	98.972
	uf6	44	1.028	1.028	100.000
	Missing	0	0.000		
	Total	4280	100.000		

Frequencies for Population

Call type	Population	Frequency	Percent	Valid Percent	Cumulative Percent
Grumph	Gunung Palung	471	14.208	14.208	14.208
	Sampan Getek	377	11.373	11.373	25.581
	Tuanan	2467	74.419	74.419	100.000
	Missing	0	0.000		
	Total	3315	100.000		
Kiss Squeak	Gunung Palung	637	14.883	14.883	14.883
	Sampan Getek	484	11.308	11.308	26.192
	Tuanan	3159	73.808	73.808	100.000
	Missing	0	0.000		
	Total	4280	100.000		

Frequencies for Paradigm

Call type	Paradigm	Frequency	Percent	Valid Percent	Cumulative Percent
Grumph	Contextual	850	25.641	25.641	25.641
	Geographical	1650	49.774	49.774	75.415
	Individual	815	24.585	24.585	100.000
	Missing	0	0.000		
	Total	3315	100.000		
Kiss Squeak	Contextual	1038	24.252	24.252	24.252
	Geographical	2184	51.028	51.028	75.280
	Individual	1058	24.720	24.720	100.000
	Missing	0	0.000		
	Total	4280	100.000		

Frequencies for Context

Call type	Context	Frequency	Percent	Valid Percent	Cumulative Percent
Grumph	observers	2801	84.495	84.495	84.495
	tiger	282	8.507	8.507	93.002
	white	232	6.998	6.998	100.000
	Missing	0	0.000		
	Total	3315	100.000		
Kiss Squeak	observers	3646	85.187	85.187	85.187
	tiger	341	7.967	7.967	93.154
	white	293	6.846	6.846	100.000
	Missing	0	0.000		
	Total	4280	100.000		

Table 2. Descriptive Statistics for Population

Descriptive Statistics

	Name of Ind			Paradigm			Context		
	Gunung Palung	Sampan Getek	Tuanan	Gunung Palung	Sampan Getek	Tuanan	Gunung Palung	Sampan Getek	Tuanan
Valid	1108	861	5626	1108	861	5626	1108	861	5626
Missing	0	0	0	0	0	0	0	0	0

Frequency Tables

Frequencies for Name of Ind

Population	Name of Ind	Frequency	Percent	Valid Percent	Cumulative Percent
Gunung Palung	Aminah	55	4.964	4.964	4.964
	Asny	74	6.679	6.679	11.643
	Bintang	0	0.000	0.000	11.643
	Desy	0	0.000	0.000	11.643
	Indi	235	21.209	21.209	32.852
	Inul	0	0.000	0.000	32.852
	Jinak	0	0.000	0.000	32.852
	Juni	0	0.000	0.000	32.852
	Kasi	0	0.000	0.000	32.852
	Keri	0	0.000	0.000	32.852
	Kondor	0	0.000	0.000	32.852
	Male	0	0.000	0.000	32.852
	Mindi	0	0.000	0.000	32.852
	Pensi	0	0.000	0.000	32.852
	Sumi	0	0.000	0.000	32.852
	Walimah	196	17.690	17.690	50.542
	uf2	155	13.989	13.989	64.531
	uf3	146	13.177	13.177	77.708
	uf5	173	15.614	15.614	93.321
	uf6	74	6.679	6.679	100.000
	Missing	0	0.000		
	Total	1108	100.000		
Sampan Getek	Aminah	0	0.000	0.000	0.000
	Asny	0	0.000	0.000	0.000
	Bintang	266	30.894	30.894	30.894
	Desy	0	0.000	0.000	30.894
	Indi	0	0.000	0.000	30.894
	Inul	0	0.000	0.000	30.894
	Jinak	0	0.000	0.000	30.894
	Juni	0	0.000	0.000	30.894
	Kasi	231	26.829	26.829	57.724
	Keri	0	0.000	0.000	57.724
	Kondor	0	0.000	0.000	57.724
	Male	162	18.815	18.815	76.539
	Mindi	0	0.000	0.000	76.539
	Pensi	202	23.461	23.461	100.000
	Sumi	0	0.000	0.000	100.000
	Walimah	0	0.000	0.000	100.000
	uf2	0	0.000	0.000	100.000
	uf3	0	0.000	0.000	100.000
	uf5	0	0.000	0.000	100.000
	uf6	0	0.000	0.000	100.000
	Missing	0	0.000		
	Total	861	100.000		

Tuanan	Aminah	0	0.000	0.000	0.000
	Asny	0	0.000	0.000	0.000
	Bintang	0	0.000	0.000	0.000
	Desy	1123	19.961	19.961	19.961
	Indi	0	0.000	0.000	19.961
	Inul	769	13.669	13.669	33.630
	Jinak	703	12.496	12.496	46.125
	Juni	1059	18.823	18.823	64.948
	Kasi	0	0.000	0.000	64.948
	Keri	947	16.833	16.833	81.781
	Kondor	367	6.523	6.523	88.304
	Male	0	0.000	0.000	88.304
	Mindi	280	4.977	4.977	93.281
	Pensi	0	0.000	0.000	93.281
	Sumi	378	6.719	6.719	100.000
	Walimah	0	0.000	0.000	100.000
	uf2	0	0.000	0.000	100.000
	uf3	0	0.000	0.000	100.000
	uf5	0	0.000	0.000	100.000
	uf6	0	0.000	0.000	100.000
	Missing	0	0.000		
	Total	5626	100.000		

Frequencies for Paradigm

Population	Paradigm	Frequency	Percent	Valid Percent	Cumulative Percent
Gunung Palung	Contextual	0	0.000	0.000	0.000
	Geographical	1108	100.000	100.000	100.000
	Individual	0	0.000	0.000	100.000
	Missing	0	0.000		
	Total	1108	100.000		
Sampan Getek	Contextual	0	0.000	0.000	0.000
	Geographical	861	100.000	100.000	100.000
	Individual	0	0.000	0.000	100.000
	Missing	0	0.000		
	Total	861	100.000		
Tuanan	Contextual	1888	33.558	33.558	33.558
	Geographical	1865	33.150	33.150	66.708
	Individual	1873	33.292	33.292	100.000
	Missing	0	0.000		
	Total	5626	100.000		

Frequencies for Context

Population	Context	Frequency	Percent	Valid Percent	Cumulative Percent
Gunung Palung	observers	1108	100.000	100.000	100.000
	tiger	0	0.000	0.000	100.000
	white	0	0.000	0.000	100.000
	Missing	0	0.000		
	Total	1108	100.000		
Sampan Getek	observers	861	100.000	100.000	100.000
	tiger	0	0.000	0.000	100.000
	white	0	0.000	0.000	100.000
	Missing	0	0.000		
	Total	861	100.000		
Tuanan	observers	4478	79.595	79.595	79.595
	tiger	623	11.074	11.074	90.668
	white	525	9.332	9.332	100.000
	Missing	0	0.000		
	Total	5626	100.000		

Table 3. Descriptive Statistics for Context

Descriptive Statistics

	Name of Ind			Population		
	observers	tiger	white	observers	tiger	white
Valid	6447	623	525	6447	623	525
Missing	0	0	0	0	0	0

Frequency Tables

Frequencies for Name of Ind

Context	Name of Ind	Frequency	Percent	Valid Percent	Cumulative Percent
observers	Aminah	55	0.853	0.853	0.853
	Asny	74	1.148	1.148	2.001
	Bintang	266	4.126	4.126	6.127
	Desy	1123	17.419	17.419	23.546
	Indi	235	3.645	3.645	27.191
	Inul	769	11.928	11.928	39.119
	Jinak	431	6.685	6.685	45.804
	Juni	847	13.138	13.138	58.942
	Kasi	231	3.583	3.583	62.525
	Keri	565	8.764	8.764	71.289
	Kondor	365	5.662	5.662	76.951
	Male	162	2.513	2.513	79.463
	Mindi	0	0.000	0.000	79.463
	Pensi	202	3.133	3.133	82.597
	Sumi	378	5.863	5.863	88.460
	Walimah	196	3.040	3.040	91.500
	uf2	155	2.404	2.404	93.904
	uf3	146	2.265	2.265	96.169
	uf5	173	2.683	2.683	98.852
	uf6	74	1.148	1.148	100.000
	Missing	0	0.000		
	Total	6447	100.000		
tiger	Aminah	0	0.000	0.000	0.000
	Asny	0	0.000	0.000	0.000
	Bintang	0	0.000	0.000	0.000
	Desy	0	0.000	0.000	0.000
	Indi	0	0.000	0.000	0.000
	Inul	0	0.000	0.000	0.000
	Jinak	128	20.546	20.546	20.546
	Juni	178	28.571	28.571	49.117
	Kasi	0	0.000	0.000	49.117
	Keri	175	28.090	28.090	77.207
	Kondor	0	0.000	0.000	77.207
	Male	0	0.000	0.000	77.207
	Mindi	142	22.793	22.793	100.000
	Pensi	0	0.000	0.000	100.000
	Sumi	0	0.000	0.000	100.000
	Walimah	0	0.000	0.000	100.000
	uf2	0	0.000	0.000	100.000
	uf3	0	0.000	0.000	100.000
	uf5	0	0.000	0.000	100.000
	uf6	0	0.000	0.000	100.000
	Missing	0	0.000		
	Total	623	100.000		

white	Aminah	0	0.000	0.000	0.000
	Asny	0	0.000	0.000	0.000
	Bintang	0	0.000	0.000	0.000
	Desy	0	0.000	0.000	0.000
	Indi	0	0.000	0.000	0.000
	Inul	0	0.000	0.000	0.000
	Jinak	144	27.429	27.429	27.429
	Juni	34	6.476	6.476	33.905
	Kasi	0	0.000	0.000	33.905
	Keri	207	39.429	39.429	73.333
	Kondor	2	0.381	0.381	73.714
	Male	0	0.000	0.000	73.714
	Mindi	138	26.286	26.286	100.000
	Pensi	0	0.000	0.000	100.000
	Sumi	0	0.000	0.000	100.000
	Walimah	0	0.000	0.000	100.000
	uf2	0	0.000	0.000	100.000
	uf3	0	0.000	0.000	100.000
	uf5	0	0.000	0.000	100.000
	uf6	0	0.000	0.000	100.000
	Missing	0	0.000		
	Total	525	100.000		

Frequencies for Population

Context	Population	Frequency	Percent	Valid Percent	Cumulative Percent
observers	Gunung Palung	1108	17.186	17.186	17.186
	Sampan Getek	861	13.355	13.355	30.541
	Tuanan	4478	69.459	69.459	100.000
	Missing	0	0.000		
	Total	6447	100.000		
tiger	Gunung Palung	0	0.000	0.000	0.000
	Sampan Getek	0	0.000	0.000	0.000
	Tuanan	623	100.000	100.000	100.000
	Missing	0	0.000		
	Total	623	100.000		
white	Gunung Palung	0	0.000	0.000	0.000
	Sampan Getek	0	0.000	0.000	0.000
	Tuanan	525	100.000	100.000	100.000
	Missing	0	0.000		
	Total	525	100.000		

Table 4. Descriptive Statistics for Distance

Descriptive Statistics

	Name of Ind									Population								
	0	25	50	75	100	150	200	300	400	0	25	50	75	100	150	200	300	400
Valid	974	974	967	954	940	920	810	573	483	974	974	967	954	940	920	810	573	483
Missing	0	0	0	0	0	0	0	0	0	0	0	0	0	0	0	0	0	0

	Context								
	0	25	50	75	100	150	200	300	400
Valid	974	974	967	954	940	920	810	573	483
Missing	0	0	0	0	0	0	0	0	0

Frequencies for Name of Ind

Distance mic-to-focal (m)	Name of Ind	Frequency	Percent	Valid Percent	Cumulative Percent
0	Aminah	8	0.821	0.821	0.821
	Asny	10	1.027	1.027	1.848
	Bintang	34	3.491	3.491	5.339
	Desy	142	14.579	14.579	19.918
	Indi	30	3.080	3.080	22.998
	Inul	96	9.856	9.856	32.854
	Jinak	92	9.446	9.446	42.300
	Juni	132	13.552	13.552	55.852
	Kasi	28	2.875	2.875	58.727
	Keri	120	12.320	12.320	71.047
	Kondor	46	4.723	4.723	75.770
	Male	20	2.053	2.053	77.823
	Mindi	36	3.696	3.696	81.520
	Pensi	26	2.669	2.669	84.189
	Sumi	52	5.339	5.339	89.528
	Walimah	30	3.080	3.080	92.608
	uf2	20	2.053	2.053	94.661
	uf3	20	2.053	2.053	96.715
	uf5	22	2.259	2.259	98.973
	uf6	10	1.027	1.027	100.000
	Missing	0	0.000		
	Total	974	100.000		
25	Aminah	8	0.821	0.821	0.821
	Asny	10	1.027	1.027	1.848
	Bintang	34	3.491	3.491	5.339
	Desy	142	14.579	14.579	19.918
	Indi	30	3.080	3.080	22.998
	Inul	96	9.856	9.856	32.854
	Jinak	92	9.446	9.446	42.300
	Juni	132	13.552	13.552	55.852
	Kasi	28	2.875	2.875	58.727
	Keri	120	12.320	12.320	71.047
	Kondor	46	4.723	4.723	75.770
	Male	20	2.053	2.053	77.823
	Mindi	36	3.696	3.696	81.520
	Pensi	26	2.669	2.669	84.189
	Sumi	52	5.339	5.339	89.528
	Walimah	30	3.080	3.080	92.608
	uf2	20	2.053	2.053	94.661
	uf3	20	2.053	2.053	96.715
	uf5	22	2.259	2.259	98.973
	uf6	10	1.027	1.027	100.000
	Missing	0	0.000		
	Total	974	100.000		

50	Aminah	8	0.827	0.827	0.827
	Asny	10	1.034	1.034	1.861
	Bintang	34	3.516	3.516	5.377
	Desy	142	14.685	14.685	20.062
	Indi	29	2.999	2.999	23.061
	Inul	96	9.928	9.928	32.989
	Jinak	92	9.514	9.514	42.503
	Juni	129	13.340	13.340	55.843
	Kasi	28	2.896	2.896	58.738
	Keri	118	12.203	12.203	70.941
	Kondor	46	4.757	4.757	75.698
	Male	20	2.068	2.068	77.766
	Mindi	36	3.723	3.723	81.489
	Pensi	26	2.689	2.689	84.178
	Sumi	52	5.377	5.377	89.555
	Walimah	30	3.102	3.102	92.658
	uf2	20	2.068	2.068	94.726
	uf3	19	1.965	1.965	96.691
	uf5	22	2.275	2.275	98.966
	uf6	10	1.034	1.034	100.000
	Missing	0	0.000		
	Total	967	100.000		
75	Aminah	7	0.734	0.734	0.734
	Asny	9	0.943	0.943	1.677
	Bintang	34	3.564	3.564	5.241
	Desy	141	14.780	14.780	20.021
	Indi	29	3.040	3.040	23.061
	Inul	96	10.063	10.063	33.124
	Jinak	86	9.015	9.015	42.138
	Juni	129	13.522	13.522	55.660
	Kasi	28	2.935	2.935	58.595
	Keri	117	12.264	12.264	70.860
	Kondor	46	4.822	4.822	75.681
	Male	20	2.096	2.096	77.778
	Mindi	35	3.669	3.669	81.447
	Pensi	26	2.725	2.725	84.172
	Sumi	50	5.241	5.241	89.413
	Walimah	30	3.145	3.145	92.558
	uf2	20	2.096	2.096	94.654
	uf3	19	1.992	1.992	96.646
	uf5	22	2.306	2.306	98.952
	uf6	10	1.048	1.048	100.000
	Missing	0	0.000		
	Total	954	100.000		
100	Aminah	7	0.745	0.745	0.745
	Asny	9	0.957	0.957	1.702
	Bintang	32	3.404	3.404	5.106

Frequencies for Name of Ind

Distance mic-to-focal (m)	Name of Ind	Frequency	Percent	Valid Percent	Cumulative Percent
	Desy	138	14.681	14.681	19.787
	Indi	27	2.872	2.872	22.660
	Inul	95	10.106	10.106	32.766
	Jinak	86	9.149	9.149	41.915
	Juni	129	13.723	13.723	55.638
	Kasi	28	2.979	2.979	58.617
	Keri	117	12.447	12.447	71.064
	Kondor	46	4.894	4.894	75.957
	Male	19	2.021	2.021	77.979
	Mindi	35	3.723	3.723	81.702
	Pensi	24	2.553	2.553	84.255
	Sumi	48	5.106	5.106	89.362
	Walimah	29	3.085	3.085	92.447
	uf2	20	2.128	2.128	94.574
	uf3	19	2.021	2.021	96.596
	uf5	22	2.340	2.340	98.936
	uf6	10	1.064	1.064	100.000
	Missing	0	0.000		
	Total	940	100.000		
150	Aminah	7	0.761	0.761	0.761
	Asny	9	0.978	0.978	1.739
	Bintang	32	3.478	3.478	5.217
	Desy	135	14.674	14.674	19.891
	Indi	27	2.935	2.935	22.826
	Inul	94	10.217	10.217	33.043
	Jinak	85	9.239	9.239	42.283
	Juni	124	13.478	13.478	55.761
	Kasi	27	2.935	2.935	58.696
	Keri	117	12.717	12.717	71.413
	Kondor	46	5.000	5.000	76.413
	Male	18	1.957	1.957	78.370
	Mindi	33	3.587	3.587	81.957
	Pensi	23	2.500	2.500	84.457
	Sumi	46	5.000	5.000	89.457
	Walimah	26	2.826	2.826	92.283
	uf2	20	2.174	2.174	94.457
	uf3	19	2.065	2.065	96.522
	uf5	22	2.391	2.391	98.913
	uf6	10	1.087	1.087	100.000
	Missing	0	0.000		
	Total	920	100.000		
200	Aminah	7	0.864	0.864	0.864
	Asny	7	0.864	0.864	1.728
	Bintang	26	3.210	3.210	4.938
	Desy	108	13.333	13.333	18.272
	Indi	23	2.840	2.840	21.111
	Inul	87	10.741	10.741	31.852
	Jinak	85	10.494	10.494	42.346
	Juni	123	15.185	15.185	57.531
	Kasi	24	2.963	2.963	60.494
	Keri	111	13.704	13.704	74.198
	Kondor	41	5.062	5.062	79.259
	Male	17	2.099	2.099	81.358
	Mindi	33	4.074	4.074	85.432
	Pensi	23	2.840	2.840	88.272
	Sumi	27	3.333	3.333	91.605
	Walimah	18	2.222	2.222	93.827
	uf2	12	1.481	1.481	95.309
	uf3	15	1.852	1.852	97.160
	uf5	18	2.222	2.222	99.383
	uf6	5	0.617	0.617	100.000
	Missing	0	0.000		
	Total	810	100.000		
300	Aminah	2	0.349	0.349	0.349
	Asny	5	0.873	0.873	1.222
	Bintang	20	3.490	3.490	4.712
	Desy	97	16.928	16.928	21.640
	Indi	20	3.490	3.490	25.131
	Inul	63	10.995	10.995	36.126
	Jinak	47	8.202	8.202	44.328
	Juni	86	15.009	15.009	59.337
	Kasi	20	3.490	3.490	62.827
	Keri	68	11.867	11.867	74.695
	Kondor	35	6.108	6.108	80.803
	Male	14	2.443	2.443	83.246
	Mindi	18	3.141	3.141	86.387
	Pensi	14	2.443	2.443	88.831
	Sumi	26	4.538	4.538	93.368
	Walimah	2	0.349	0.349	93.717
	uf2	12	2.094	2.094	95.812
	uf3	8	1.396	1.396	97.208
	uf5	11	1.920	1.920	99.127
	uf6	5	0.873	0.873	100.000
	Missing	0	0.000		
	Total	573	100.000		
400	Aminah	1	0.207	0.207	0.207
	Asny	5	1.035	1.035	1.242
	Bintang	20	4.141	4.141	5.383
	Desy	78	16.149	16.149	21.532
	Indi	20	4.141	4.141	25.673
	Inul	46	9.524	9.524	35.197

Frequencies for Name of Ind

Distance mic-to-focal (m)	Name of Ind	Frequency	Percent	Valid Percent	Cumulative Percent
	Jinak	38	7.867	7.867	43.064
	Juni	75	15.528	15.528	58.592
	Kasi	20	4.141	4.141	62.733
	Keri	59	12.215	12.215	74.948
	Kondor	15	3.106	3.106	78.054
	Male	14	2.899	2.899	80.952
	Mindi	18	3.727	3.727	84.679
	Pensi	14	2.899	2.899	87.578
	Sumi	25	5.176	5.176	92.754
	Walimah	1	0.207	0.207	92.961
	uf2	11	2.277	2.277	95.238
	uf3	7	1.449	1.449	96.687
	uf5	12	2.484	2.484	99.172
	uf6	4	0.828	0.828	100.000
	Missing	0	0.000		
	Total	483	100.000		

Frequencies for Population

Distance mic-to-focal (m)	Population	Frequency	Percent	Valid Percent	Cumulative Percent
0	Gunung Palung	150	15.400	15.400	15.400
	Sampan Getek	108	11.088	11.088	26.489
	Tuanan	716	73.511	73.511	100.000
	Missing	0	0.000		
	Total	974	100.000		
25	Gunung Palung	150	15.400	15.400	15.400
	Sampan Getek	108	11.088	11.088	26.489
	Tuanan	716	73.511	73.511	100.000
	Missing	0	0.000		
	Total	974	100.000		
50	Gunung Palung	148	15.305	15.305	15.305
	Sampan Getek	108	11.169	11.169	26.474
	Tuanan	711	73.526	73.526	100.000
	Missing	0	0.000		
	Total	967	100.000		
75	Gunung Palung	146	15.304	15.304	15.304
	Sampan Getek	108	11.321	11.321	26.625
	Tuanan	700	73.375	73.375	100.000
	Missing	0	0.000		
	Total	954	100.000		
100	Gunung Palung	143	15.213	15.213	15.213
	Sampan Getek	103	10.957	10.957	26.170
	Tuanan	694	73.830	73.830	100.000
	Missing	0	0.000		
	Total	940	100.000		
150	Gunung Palung	140	15.217	15.217	15.217
	Sampan Getek	100	10.870	10.870	26.087
	Tuanan	680	73.913	73.913	100.000
	Missing	0	0.000		
	Total	920	100.000		
200	Gunung Palung	105	12.963	12.963	12.963
	Sampan Getek	90	11.111	11.111	24.074
	Tuanan	615	75.926	75.926	100.000
	Missing	0	0.000		
	Total	810	100.000		
300	Gunung Palung	65	11.344	11.344	11.344
	Sampan Getek	68	11.867	11.867	23.211
	Tuanan	440	76.789	76.789	100.000
	Missing	0	0.000		
	Total	573	100.000		
400	Gunung Palung	61	12.629	12.629	12.629
	Sampan Getek	68	14.079	14.079	26.708
	Tuanan	354	73.292	73.292	100.000
	Missing	0	0.000		
	Total	483	100.000		

Frequencies for Context

Distance mic-to-focal (m)	Context	Frequency	Percent	Valid Percent	Cumulative Percent
0	observers	830	85.216	85.216	85.216
	tiger	76	7.803	7.803	93.018
	white	68	6.982	6.982	100.000
	Missing	0	0.000		
	Total	974	100.000		
25	observers	830	85.216	85.216	85.216
	tiger	75	7.700	7.700	92.916
	white	69	7.084	7.084	100.000
	Missing	0	0.000		
	Total	974	100.000		
50	observers	824	85.212	85.212	85.212
	tiger	76	7.859	7.859	93.071
	white	67	6.929	6.929	100.000
	Missing	0	0.000		
	Total	967	100.000		
75	observers	811	85.010	85.010	85.010
	tiger	75	7.862	7.862	92.872
	white	68	7.128	7.128	100.000
	Missing	0	0.000		
	Total	954	100.000		
100	observers	801	85.213	85.213	85.213
	tiger	76	8.085	8.085	93.298
	white	63	6.702	6.702	100.000
	Missing	0	0.000		
	Total	940	100.000		
150	observers	784	85.217	85.217	85.217
	tiger	76	8.261	8.261	93.478
	white	60	6.522	6.522	100.000
	Missing	0	0.000		
	Total	920	100.000		
200	observers	674	83.210	83.210	83.210
	tiger	74	9.136	9.136	92.346
	white	62	7.654	7.654	100.000
	Missing	0	0.000		
	Total	810	100.000		
300	observers	490	85.515	85.515	85.515
	tiger	48	8.377	8.377	93.892
	white	35	6.108	6.108	100.000
	Missing	0	0.000		
	Total	573	100.000		
400	observers	403	83.437	83.437	83.437
	tiger	47	9.731	9.731	93.168
	white	33	6.832	6.832	100.000
	Missing	0	0.000		
	Total	483	100.000		
