# Supplementary material for: Open plains are not a level playing field for hominid consonant-like versus vowel-like calls
Source: Sci Rep. 2023 Dec 21;13:21138. doi: 10.1038/s41598-023-48165-7 (PMC10739746; doi:10.1038/s41598-023-48165-7)
Supplement: Supplementary file 2 — Supplementary Information 2. [file 41598_2023_48165_MOESM2_ESM.pdf]

# Results

## KSQ_Delta Time_Linear Mixed Models

ANOVA Summary

| Effect | df | F | p |
|---|---|---|---|
| Distance mic-to-focal (m) | 8, 4215.78 | 18.593 | < .001 |
| Context | 2, 3035.90 | 25.784 | < .001 |
| Population | 2, 17.01 | 4.579 | 0.026 |

*Note.* Model terms tested with Satterthwaite method.
*Note.* The following variables are used as random effects grouping factors: 'Name of Ind', 'Call ID'.
*Note.* Type III Sum of Squares

### Model summary

Fit statistics

| Deviance (REML) | log Lik. | df | AIC | BIC |
|---|---|---|---|---|
| −5188.834 | 2594.417 | 16 | −5156.834 | −5055.054 |

*Note.* The model was fitted using restricted maximum likelihood. Please note that models with different fixed effects cannot be compared when REML is used. To use ML, switch 'Test model terms' to 'Likelihood ratio tests'.

Sample sizes

| | Levels of RE grouping factors | |
|---|---|---|
| Observations | Call ID | Name of Ind |
| 4278 | 33 | 20 |

Fixed Effects Estimates

| Term | Estimate | SE | df | t | p |
|---|---|---|---|---|---|
| Intercept | 0.476 | 0.027 | 19.375 | 17.939 | < .001 |
| Distance mic-to-focal (m) (1) | 0.011 | 0.005 | 4214.566 | 2.058 | 0.040 |
| Distance mic-to-focal (m) (2) | 0.029 | 0.005 | 4214.559 | 5.266 | < .001 |
| Distance mic-to-focal (m) (3) | $1.357 \times 10^{-4}$ | 0.005 | 4214.548 | 0.025 | 0.980 |
| Distance mic-to-focal (m) (4) | 0.018 | 0.005 | 4215.999 | 3.245 | 0.001 |
| Distance mic-to-focal (m) (5) | −0.003 | 0.005 | 4214.683 | −0.631 | 0.528 |
| Distance mic-to-focal (m) (6) | −0.045 | 0.005 | 4215.659 | −8.192 | < .001 |
| Distance mic-to-focal (m) (7) | −0.033 | 0.006 | 4214.614 | −6.026 | < .001 |
| Distance mic-to-focal (m) (8) | −0.003 | 0.006 | 4216.332 | −0.582 | 0.561 |
| Context (1) | −0.011 | 0.005 | 2911.630 | −2.093 | 0.036 |
| Context (2) | 0.041 | 0.006 | 4095.046 | 7.181 | < .001 |
| Population (1) | 0.033 | 0.034 | 17.141 | 0.975 | 0.343 |
| Population (2) | 0.069 | 0.041 | 16.982 | 1.710 | 0.106 |

*Note.* The intercept corresponds to the (unweighted) grand mean; for each factor with k levels, k - 1 parameters are estimated with sum contrast coding. Consequently, the estimates cannot be directly mapped to factor levels. Use estimated marginal means for obtaining estimates for each factor level/design cell or their differences.

Estimated Marginal Means

| | | | 95% CI | |
|---|---|---|---|---|
| Population | Estimate | SE | Lower | Upper |
| Gunung Palung | 0.509 | 0.039 | 0.432 | 0.586 |
| Sampan Getek | 0.545 | 0.055 | 0.438 | 0.653 |
| Tuanan | 0.373 | 0.039 | 0.297 | 0.450 |

*Note.* Results are averaged over the levels of: Distance mic-to-focal (m), Context.

# KSQ_Max Freq_Linear Mixed Models

ANOVA Summary

| Effect | df | F | p |
|---|---|---|---|
| Distance mic-to-focal (m) | 8, 4193.96 | 17.194 | < .001 |
| Context | 2, 1530.42 | 21.084 | < .001 |
| Population | 2, 16.83 | 1.013 | 0.384 |

*Note.* Model terms tested with Satterthwaite method.
*Note.* The following variables are used as random effects grouping factors: 'Name of Ind', 'Call ID'.
*Note.* Type III Sum of Squares

## Model summary

Fit statistics

| Deviance (REML) | log Lik. | df | AIC | BIC |
|---|---|---|---|---|
| 67143.176 | −33571.588 | 16 | 67175.176 | 67276.956 |

*Note.* The model was fitted using restricted maximum likelihood. Please note that models with different fixed effects cannot be compared when REML is used. To use ML, switch 'Test model terms' to 'Likelihood ratio tests'.

Sample sizes

| | Levels of RE grouping factors | |
|---|---|---|
| Observations | Call ID | Name of Ind |
| 4278 | 33 | 20 |

Fixed Effects Estimates

| Term | Estimate | SE | df | t | p |
|---|---|---|---|---|---|
| Intercept | 3608.005 | 106.968 | 18.971 | 33.730 | < .001 |
| Distance mic–to–focal (m) (1) | 66.486 | 26.537 | 4191.705 | 2.505 | 0.012 |
| Distance mic–to–focal (m) (2) | −84.201 | 26.537 | 4191.669 | −3.173 | 0.002 |
| Distance mic–to–focal (m) (3) | 50.979 | 26.537 | 4191.703 | 1.921 | 0.055 |
| Distance mic–to–focal (m) (4) | 85.670 | 26.538 | 4193.223 | 3.228 | 0.001 |
| Distance mic–to–focal (m) (5) | 116.976 | 26.538 | 4192.025 | 4.408 | < .001 |
| Distance mic–to–focal (m) (6) | 49.562 | 26.539 | 4193.269 | 1.868 | 0.062 |
| Distance mic–to–focal (m) (7) | 58.637 | 26.584 | 4191.845 | 2.206 | 0.027 |
| Distance mic–to–focal (m) (8) | −71.211 | 27.398 | 4195.110 | −2.599 | 0.009 |
| Context (1) | 123.868 | 25.477 | 1321.210 | 4.862 | < .001 |
| Context (2) | −152.391 | 27.236 | 3568.972 | −5.595 | < .001 |
| Population (1) | −91.317 | 137.661 | 17.002 | −0.663 | 0.516 |
| Population (2) | −99.984 | 163.989 | 16.782 | −0.610 | 0.550 |

*Note.* The intercept corresponds to the (unweighted) grand mean; for each factor with k levels, k – 1 parameters are estimated with sum contrast coding. Consequently, the estimates cannot be directly mapped to factor levels. Use estimated marginal means for obtaining estimates for each factor level/design cell or their differences.

Estimated Marginal Means

| | | | 95% CI | |
|---|---|---|---|---|
| Population | Estimate | SE | Lower | Upper |
| Gunung Palung | 3516.688 | 159.221 | 3204.621 | 3828.756 |
| Sampan Getek | 3508.021 | 221.777 | 3073.346 | 3942.696 |
| Tuanan | 3799.305 | 156.207 | 3493.145 | 4105.466 |

*Note.* Results are averaged over the levels of: Distance mic–to–focal (m), Context.

# KSQ_Max Slope_Linear Mixed Models

ANOVA Summary

| Effect | df | F | p |
|---|---|---|---|
| Distance mic-to-focal (m) | 8, 4208.45 | 58.692 | < .001 |
| Context | 2, 2199.88 | 11.328 | < .001 |
| Population | 2, 16.94 | 1.684 | 0.215 |

*Note.* Model terms tested with Satterthwaite method.

*Note.* The following variables are used as random effects grouping factors: 'Name of Ind', 'Call ID'.

*Note.* Type III Sum of Squares

## Model summary

Fit statistics

| Deviance (REML) | log Lik. | df | AIC | BIC |
|---|---|---|---|---|
| 47351.924 | −23675.962 | 16 | 47383.924 | 47485.704 |

*Note.* The model was fitted using restricted maximum likelihood. Please note that models with different fixed effects cannot be compared when REML is used. To use ML, switch 'Test model terms' to 'Likelihood ratio tests'.

Sample sizes

| | Levels of RE grouping factors | |
|---|---|---|
| Observations | Call ID | Name of Ind |
| 4278 | 33 | 20 |

Fixed Effects Estimates

| Term | Estimate | SE | df | t | p |
|---|---|---|---|---|---|
| Intercept | 104.754 | 5.844 | 27.199 | 17.927 | < .001 |
| Distance mic-to-focal (m) (1) | 35.676 | 2.612 | 4206.544 | 13.658 | < .001 |
| Distance mic-to-focal (m) (2) | 25.235 | 2.612 | 4206.527 | 9.661 | < .001 |
| Distance mic-to-focal (m) (3) | −4.840 | 2.612 | 4206.532 | −1.853 | 0.064 |
| Distance mic-to-focal (m) (4) | −4.775 | 2.612 | 4207.932 | −1.828 | 0.068 |
| Distance mic-to-focal (m) (5) | −15.944 | 2.612 | 4206.743 | −6.104 | < .001 |
| Distance mic-to-focal (m) (6) | −24.094 | 2.612 | 4207.784 | −9.224 | < .001 |
| Distance mic-to-focal (m) (7) | −22.660 | 2.617 | 4206.585 | −8.660 | < .001 |
| Distance mic-to-focal (m) (8) | 16.831 | 2.697 | 4209.777 | 6.241 | < .001 |
| Context (1) | −11.583 | 2.513 | 1968.425 | −4.610 | < .001 |
| Context (2) | 0.606 | 2.690 | 3881.903 | 0.225 | 0.822 |
| Population (1) | 12.601 | 6.881 | 17.616 | 1.831 | 0.084 |
| Population (2) | −8.229 | 8.123 | 16.819 | −1.013 | 0.325 |

*Note.* The intercept corresponds to the (unweighted) grand mean; for each factor with k levels, k − 1 parameters are estimated with sum contrast coding. Consequently, the estimates cannot be directly mapped to factor levels. Use estimated marginal means for obtaining estimates for each factor level/design cell or their differences.

Estimated Marginal Means

| | | | 95% CI | |
|---|---|---|---|---|
| Population | Estimate | SE | Lower | Upper |
| Gunung Palung | 117.355 | 8.454 | 100.785 | 133.925 |
| Sampan Getek | 96.525 | 11.290 | 74.397 | 118.653 |
| Tuanan | 100.382 | 7.904 | 84.891 | 115.874 |

*Note.* Results are averaged over the levels of: Distance mic-to-focal (m), Context.

# KSQ_SNR_Linear Mixed Models

ANOVA Summary

| Effect | df | F | p |
|---|---|---|---|
| Distance mic-to-focal (m) | 8, 4204.24 | 358.554 | < .001 |
| Context | 2, 3568.54 | 3.307 | 0.037 |
| Population | 2, 16.72 | 8.532 | 0.003 |

*Note.* Model terms tested with Satterthwaite method.
*Note.* The following variables are used as random effects grouping factors: 'Name of Ind', 'Call ID'.
*Note.* Type III Sum of Squares

## Model summary

Fit statistics

| Deviance (REML) | log Lik. | df | AIC | BIC |
|---|---|---|---|---|
| 18821.121 | −9410.561 | 16 | 18853.121 | 18954.901 |

*Note.* The model was fitted using restricted maximum likelihood. Please note that models with different fixed effects cannot be compared when REML is used. To use ML, switch 'Test model terms' to 'Likelihood ratio tests'.

Sample sizes

| | Levels of RE grouping factors | |
|---|---|---|
| Observations | Call ID | Name of Ind |
| 4278 | 33 | 20 |

Fixed Effects Estimates

| Term | Estimate | SE | df | t | p |
|---|---|---|---|---|---|
| Intercept | 17.087 | 0.318 | 25.248 | 53.809 | < .001 |
| Distance mic-to-focal (m) (1) | 1.500 | 0.092 | 4202.768 | 16.367 | < .001 |
| Distance mic-to-focal (m) (2) | 2.349 | 0.092 | 4202.765 | 25.638 | < .001 |
| Distance mic-to-focal (m) (3) | 1.309 | 0.092 | 4202.747 | 14.291 | < .001 |
| Distance mic-to-focal (m) (4) | 1.221 | 0.092 | 4205.240 | 13.324 | < .001 |
| Distance mic-to-focal (m) (5) | 0.584 | 0.092 | 4202.858 | 6.375 | < .001 |
| Distance mic-to-focal (m) (6) | −0.400 | 0.092 | 4204.473 | −4.367 | < .001 |
| Distance mic-to-focal (m) (7) | −0.762 | 0.092 | 4202.791 | −8.303 | < .001 |
| Distance mic-to-focal (m) (8) | −2.477 | 0.095 | 4204.834 | −26.184 | < .001 |
| Context (1) | −0.223 | 0.091 | 3499.263 | −2.443 | 0.015 |
| Context (2) | 0.135 | 0.095 | 4202.580 | 1.416 | 0.157 |
| Population (1) | −1.550 | 0.377 | 17.003 | −4.109 | < .001 |
| Population (2) | 0.956 | 0.448 | 16.657 | 2.132 | 0.048 |

*Note.* The intercept corresponds to the (unweighted) grand mean; for each factor with k levels, k − 1 parameters are estimated with sum contrast coding. Consequently, the estimates cannot be directly mapped to factor levels. Use estimated marginal means for obtaining estimates for each factor level/design cell or their differences.

Estimated Marginal Means

| | | | 95% CI | |
|---|---|---|---|---|
| Population | Estimate | SE | Lower | Upper |
| Gunung Palung | 15.537 | 0.455 | 14.646 | 16.429 |
| Sampan Getek | 18.043 | 0.619 | 16.829 | 19.256 |
| Tuanan | 17.681 | 0.442 | 16.814 | 18.547 |

*Note.* Results are averaged over the levels of: Distance mic-to-focal (m), Context.

# KSQ_Max Amp_Linear Mixed Models

ANOVA Summary

| Effect | df | F | p |
|---|---|---|---|
| Distance mic-to-focal (m) | 8, 4248.36 | 2301.855 | < .001 |
| Context | 2, 3563.30 | 15.398 | < .001 |
| Population | 2, 16.47 | 0.415 | 0.667 |

Warning: Model fit is singular. Specified random effects parameters (random intercepts and random slopes) cannot be estimated from the available data. Carefully reduce the random effects structure, but this practice might inflate the reported p-value, and invalidates the analysis.

*Note.* Model terms tested with Satterthwaite method.

*Note.* The following variables are used as random effects grouping factors: 'Name of Ind', 'Call ID'.

*Note.* Type III Sum of Squares

## Model summary

Fit statistics

| Deviance (REML) | log Lik. | df | AIC | BIC |
|---|---|---|---|---|
| 84201.478 | −42100.739 | 16 | 84233.478 | 84335.258 |

*Note.* The model was fitted using restricted maximum likelihood. Please note that models with different fixed effects cannot be compared when REML is used. To use ML, switch 'Test model terms' to 'Likelihood ratio tests'.

Sample sizes

| | Levels of RE grouping factors | |
|---|---|---|
| Observations | Call ID | Name of Ind |
| 4278 | 33 | 20 |

Fixed Effects Estimates

| Term | Estimate | SE | df | t | p |
|---|---|---|---|---|---|
| Intercept | 8961.999 | 325.107 | 26.093 | 27.566 | < .001 |
| Distance mic-to-focal (m) (1) | 20498.027 | 197.585 | 4247.702 | 103.743 | < .001 |
| Distance mic-to-focal (m) (2) | 14062.155 | 197.585 | 4247.702 | 71.170 | < .001 |
| Distance mic-to-focal (m) (3) | −1431.307 | 197.585 | 4247.702 | −7.244 | < .001 |
| Distance mic-to-focal (m) (4) | −5358.012 | 197.588 | 4247.713 | −27.117 | < .001 |
| Distance mic-to-focal (m) (5) | −5519.929 | 197.585 | 4247.702 | −27.937 | < .001 |
| Distance mic-to-focal (m) (6) | −7286.402 | 197.588 | 4247.713 | −36.877 | < .001 |
| Distance mic-to-focal (m) (7) | −6331.030 | 197.930 | 4247.596 | −31.986 | < .001 |
| Distance mic-to-focal (m) (8) | −3447.447 | 203.948 | 4249.589 | −16.904 | < .001 |
| Context (1) | −915.931 | 172.405 | 3054.749 | −5.313 | < .001 |
| Context (2) | 664.889 | 197.461 | 4263.162 | 3.367 | < .001 |
| Population (1) | −319.955 | 388.210 | 17.588 | −0.824 | 0.421 |
| Population (2) | 369.135 | 454.685 | 16.360 | 0.812 | 0.429 |

*Note.* The intercept corresponds to the (unweighted) grand mean; for each factor with k levels, k – 1 parameters are estimated with sum contrast coding. Consequently, the estimates cannot be directly mapped to factor levels. Use estimated marginal means for obtaining estimates for each factor level/design cell or their differences.
