# Supplementary material for: Open plains are not a level playing field for hominid consonant-like versus vowel-like calls
Source: Sci Rep. 2023 Dec 21;13:21138. doi: 10.1038/s41598-023-48165-7 (PMC10739746; doi:10.1038/s41598-023-48165-7)
Supplement: Supplementary file 3 — Supplementary Information 3. [file 41598_2023_48165_MOESM3_ESM.pdf]

Results

GR_Delta Time_Linear Mixed Models

ANOVA Summary

Effect	df	F	p
Distance mic-to-focal (m)	8, 3234.13	32.474	< .001
Context	2, 2724.98	24.760	< .001
Population	2, 17.20	3.464	0.054

Note. Model terms tested with Satterthwaite testMethod.
Note. The following variables are used as random effects grouping factors: 'Name of Ind', 'Call ID'.
Note. Type III Sum of Squares

Model summary

Fit statistics

Deviance (REML)	log Lik.	df	AIC	BIC
−8258.192	4129.096	16	−8226.192	−8128.556

Note. The model was fitted using restricted maximum likelihood. Please note that models with different fixed effects cannot be compared when REML is used. To use ML, switch 'Test method' to 'Likelihood ratio tests'.

Sample sizes

	Levels of RE grouping factors	
Observations	Call ID	Name of Ind
3302	33	20

Fixed Effects Estimates

Term	Estimate	SE	df	t	p
Intercept	0.278	0.012	24.305	23.985	< .001
Distance mic-to-focal (m) (1)	0.018	0.003	3232.677	5.822	< .001
Distance mic-to-focal (m) (2)	−0.010	0.003	3232.252	−3.220	0.001
Distance mic-to-focal (m) (3)	−0.011	0.003	3232.699	−3.583	< .001
Distance mic-to-focal (m) (4)	−0.003	0.003	3235.341	−0.918	0.359
Distance mic-to-focal (m) (5)	0.018	0.003	3233.114	5.711	< .001
Distance mic-to-focal (m) (6)	−0.023	0.003	3234.036	−7.023	< .001
Distance mic-to-focal (m) (7)	−0.039	0.004	3234.292	−10.685	< .001
Distance mic-to-focal (m) (8)	0.014	0.006	3237.695	2.379	0.017
Context (1)	−0.008	0.003	2673.689	−2.575	0.010
Context (2)	0.023	0.003	3225.275	6.989	< .001
Population (1)	0.032	0.014	17.496	2.311	0.033
Population (2)	−0.006	0.017	17.116	−0.380	0.708

Note. The intercept corresponds to the (unweighted) grand mean; for each factor with k levels, k − 1 parameters are estimated with sum contrast coding. Consequently, the estimates cannot be directly mapped to factor levels. Use estimated marginal means for obtaining estimates for each factor level/design cell or their differences.

Estimated Marginal Means

				95% CI	
Population	Context	Estimate	SE	Lower	Upper
Gunung Palung	observers	0.302	0.017	0.270	0.335
Sampan Getek	observers	0.263	0.023	0.219	0.308
Tuanan	observers	0.244	0.016	0.212	0.276
Gunung Palung	tiger	0.334	0.017	0.300	0.368
Sampan Getek	tiger	0.295	0.023	0.249	0.341
Tuanan	tiger	0.275	0.017	0.242	0.308
Gunung Palung	white	0.295	0.017	0.261	0.330
Sampan Getek	white	0.257	0.023	0.211	0.303
Tuanan	white	0.237	0.017	0.204	0.270

Note. Results are averaged over the levels of: Distance mic-to-focal (m).

GR_Max Freq_Linear Mixed Models

ANOVA Summary

Effect	df	F	p
Distance mic-to-focal (m)	8, 3279.88	24.967	< .001
Context	2, 1537.06	0.553	0.575
Population	2, 20.17	0.223	0.802

Note. Model terms tested with Satterthwaite testMethod.

Note. The following variable is used as a random effects grouping factor: 'Name of Ind'.

Note. Type III Sum of Squares

Model summary

Fit statistics

Deviance (REML)	log Lik.	df	AIC	BIC
44965.148	−22482.574	15	44995.148	45086.683

Note. The model was fitted using restricted maximum likelihood. Please note that models with different fixed effects cannot be compared when REML is used. To use ML, switch 'Test method' to 'Likelihood ratio tests'.

Sample sizes

	Levels of RE grouping factors
Observations	Name of Ind
3302	20

Fixed Effects Estimates

Term	Estimate	SE	df	t	p
Intercept	275.986	12.649	55.762	21.818	< .001
Distance mic-to-focal (m) (1)	−8.903	10.179	3281.629	−0.875	0.382
Distance mic-to-focal (m) (2)	−27.498	10.179	3281.606	−2.701	0.007
Distance mic-to-focal (m) (3)	−57.378	10.235	3281.920	−5.606	< .001
Distance mic-to-focal (m) (4)	−85.375	10.337	3280.421	−8.259	< .001
Distance mic-to-focal (m) (5)	−82.565	10.459	3280.389	−7.894	< .001
Distance mic-to-focal (m) (6)	−39.717	10.645	3279.838	−3.731	< .001
Distance mic-to-focal (m) (7)	−48.420	11.983	3285.118	−4.041	< .001
Distance mic-to-focal (m) (8)	140.493	19.925	3284.148	7.051	< .001
Context (1)	7.413	8.934	979.136	0.830	0.407
Context (2)	−9.317	10.580	3232.882	−0.881	0.379
Population (1)	8.818	13.220	24.155	0.667	0.511
Population (2)	−5.988	14.966	20.432	−0.400	0.693

Note. The intercept corresponds to the (unweighted) grand mean; for each factor with k levels, k − 1 parameters are estimated with sum contrast coding. Consequently, the estimates cannot be directly mapped to factor levels. Use estimated marginal means for obtaining estimates for each factor level/design cell or their differences.

Estimated Marginal Means

			95% CI	
Population	Estimate	SE	Lower	Upper
Gunung Palung	284.804	18.503	248.539	321.069
Sampan Getek	269.998	21.986	226.906	313.091
Tuanan	273.156	14.133	245.456	300.856

Note. Results are averaged over the levels of: Distance mic-to-focal (m), Context.

GR_Max Slope_Linear Mixed Models

ANOVA Summary

Effect	df	F	p
Distance mic-to-focal (m)	8, 3257.33	24.126	< .001
Context	2, 1181.18	18.057	< .001
Population	2, 16.99	0.704	0.509

Note. Model terms tested with Satterthwaite method.
Note. The following variables are used as random effects grouping factors: 'Name of Ind', 'Call ID'.
Note. Type III Sum of Squares

Model summary

Fit statistics

Deviance (REML)	log Lik.	df	AIC	BIC
23074.313	−11537.156	16	23106.313	23203.949

Note. The model was fitted using restricted maximum likelihood. Please note that models with different fixed effects cannot be compared when REML is used. To use ML, switch 'Test model terms' to 'Likelihood ratio tests'.

Sample sizes

	Levels of RE grouping factors	
Observations	Call ID	Name of Ind
3302	33	20

Fixed Effects Estimates

Term	Estimate	SE	df	t	p
Intercept	12.928	0.668	27.406	19.356	< .001
Distance mic-to-focal (m) (1)	1.839	0.364	3255.588	5.053	< .001
Distance mic-to-focal (m) (2)	−0.903	0.364	3254.996	−2.482	0.013
Distance mic-to-focal (m) (3)	−1.318	0.366	3255.113	−3.602	< .001
Distance mic-to-focal (m) (4)	−0.005	0.370	3254.865	−0.013	0.990
Distance mic-to-focal (m) (5)	−1.294	0.374	3254.398	−3.461	< .001
Distance mic-to-focal (m) (6)	−3.058	0.381	3259.837	−8.034	< .001
Distance mic-to-focal (m) (7)	−3.383	0.429	3258.330	−7.893	< .001
Distance mic-to-focal (m) (8)	2.173	0.713	3270.170	3.049	0.002
Context (1)	−2.018	0.338	843.763	−5.968	< .001
Context (2)	1.015	0.382	2622.935	2.655	0.008
Population (1)	0.303	0.795	18.182	0.381	0.708
Population (2)	0.610	0.929	16.784	0.657	0.520

Note. The intercept corresponds to the (unweighted) grand mean; for each factor with k levels, k − 1 parameters are estimated with sum contrast coding. Consequently, the estimates cannot be directly mapped to factor levels. Use estimated marginal means for obtaining estimates for each factor level/design cell or their differences.

GR_SNR_Linear Mixed Models

ANOVA Summary

Effect	df	F	p
Distance mic-to-focal (m)	8, 3238.21	156.573	< .001
Context	2, 920.94	0.054	0.948
Population	2, 18.01	0.547	0.588

Note. Model terms tested with Satterthwaite method.
Note. The following variables are used as random effects grouping factors: 'Name of Ind', 'Call ID'.
Note. Type III Sum of Squares

Model summary

Fit statistics

Deviance (REML)	log Lik.	df	AIC	BIC
19171.550	−9585.775	16	19203.550	19301.186

Note. The model was fitted using restricted maximum likelihood. Please note that models with different fixed effects cannot be compared when REML is used. To use ML, switch 'Test model terms' to 'Likelihood ratio tests'.

Sample sizes

	Levels of RE grouping factors	
Observations	Call ID	Name of Ind
3302	33	20

Fixed Effects Estimates

Term	Estimate	SE	df	t	p
Intercept	18.783	0.416	27.131	45.174	< .001
Distance mic-to-focal (m) (1)	0.907	0.201	3234.889	4.519	< .001
Distance mic-to-focal (m) (2)	−0.380	0.201	3233.635	−1.892	0.059
Distance mic-to-focal (m) (3)	1.937	0.202	3234.000	9.599	< .001
Distance mic-to-focal (m) (4)	5.182	0.204	3234.503	25.427	< .001
Distance mic-to-focal (m) (5)	3.843	0.206	3233.846	18.637	< .001
Distance mic-to-focal (m) (6)	−1.773	0.210	3241.359	−8.447	< .001
Distance mic-to-focal (m) (7)	−2.567	0.236	3237.379	−10.861	< .001
Distance mic-to-focal (m) (8)	−1.989	0.393	3257.234	−5.058	< .001
Context (1)	−0.034	0.192	689.562	−0.177	0.860
Context (2)	0.068	0.213	2436.314	0.319	0.750
Population (1)	−0.331	0.501	18.992	−0.661	0.516
Population (2)	0.615	0.588	17.802	1.046	0.310

Note. The intercept corresponds to the (unweighted) grand mean; for each factor with k levels, k – 1 parameters are estimated with sum contrast coding. Consequently, the estimates cannot be directly mapped to factor levels. Use estimated marginal means for obtaining estimates for each factor level/design cell or their differences.

Estimated Marginal Means

			95% CI	
Population	Estimate	SE	Lower	Upper
Gunung Palung	18.451	0.615	17.246	19.656
Sampan Getek	19.398	0.813	17.804	20.992
Tuanan	18.498	0.562	17.397	19.600

Note. Results are averaged over the levels of: Distance mic-to-focal (m), Context.

GR_Max Amp_Linear Mixed Models

ANOVA Summary

Effect	df	F	p
Distance mic-to-focal (m)	8, 3238.36	591.230	< .001
Context	2, 1275.60	5.993	0.003
Population	2, 17.40	2.944	0.079

Note. Model terms tested with Satterthwaite method.
Note. The following variables are used as random effects grouping factors: 'Name of Ind', 'Call ID'.
Note. Type III Sum of Squares

Model summary

Fit statistics

Deviance (REML)	log Lik.	df	AIC	BIC
64043.374	−32021.687	16	64075.374	64173.011

Note. The model was fitted using restricted maximum likelihood. Please note that models with different fixed effects cannot be compared when REML is used. To use ML, switch 'Test model terms' to 'Likelihood ratio tests'.

Sample sizes

	Levels of RE grouping factors	
Observations	Call ID	Name of Ind
3302	33	20

Fixed Effects Estimates

Term	Estimate	SE	df	t	p
Intercept	4907.803	389.943	27.506	12.586	< .001
Distance mic-to-focal (m) (1)	10204.127	183.820	3235.891	55.511	< .001
Distance mic-to-focal (m) (2)	5361.537	183.814	3234.842	29.168	< .001
Distance mic-to-focal (m) (3)	1258.224	184.838	3235.382	6.807	< .001
Distance mic-to-focal (m) (4)	−1843.872	186.657	3236.066	−9.878	< .001
Distance mic-to-focal (m) (5)	−1877.086	188.860	3235.455	−9.939	< .001
Distance mic-to-focal (m) (6)	−3575.845	192.293	3239.890	−18.596	< .001
Distance mic-to-focal (m) (7)	−3319.964	216.493	3238.672	−15.335	< .001
Distance mic-to-focal (m) (8)	−2521.819	360.384	3251.817	−6.998	< .001
Context (1)	−624.609	180.723	1049.658	−3.456	< .001
Context (2)	246.736	196.729	2734.683	1.254	0.210
Population (1)	801.305	462.857	18.348	1.731	0.100
Population (2)	161.789	543.583	17.205	0.298	0.770

Note. The intercept corresponds to the (unweighted) grand mean; for each factor with k levels, k – 1 parameters are estimated with sum contrast coding. Consequently, the estimates cannot be directly mapped to factor levels. Use estimated marginal means for obtaining estimates for each factor level/design cell or their differences.
